# Monte Carlo-Based Optical Simulation of Optical Distribution in Deep Brain Tissues Using Sixteen Optical Sources

**DOI:** 10.3390/bioengineering11030260

**Published:** 2024-03-07

**Authors:** Xi Yang, Chengpeng Chai, Hongzhi Zuo, Yun-Hsuan Chen, Junhui Shi, Cheng Ma, Mohamad Sawan

**Affiliations:** 1 College of Biomedical Engineering & Instrument Science, Zhejiang University, 38 Zheda Road, Hangzhou 310013, China; yangxi@westlake.edu.cn; 2 CenBRAIN Neurotech Center of Excellence, School of Engineering, Westlake University, 600 Dunyu Road, Xihu District, Hangzhou 310030, China; 3 Institute of Advanced Technology, Westlake Institute for Advanced Study, 18 Shilongshan Street, Xihu District, Hangzhou 310024, China; 4 Beijing National Research Center for Information Science and Technology, Department of Electronic Engineering, Tsinghua University, 30, Shuangqing Road, Haidian District, Beijing 100084, China; 5 Zhejiang Lab, 1 Kechuang Avenue, Yuhang District, Hangzhou 311100, China

**Keywords:** photoacoustic imaging, optical imaging, brain imaging, optogenetics, functional near-infrared spectroscopy (fNIRS), optical simulation, Monte Carlo simulation

## Abstract

Optical-based imaging has improved from early single-location research to further sophisticated imaging in 2D topography and 3D tomography. These techniques have the benefit of high specificity and non-radiative safety for brain detection and therapy. However, their performance is limited by complex tissue structures. To overcome the difficulty in successful brain imaging applications, we conducted a simulation using 16 optical source types within a brain model that is based on the Monte Carlo method. In addition, we propose an evaluation method of the optical propagating depth and resolution, specifically one based on the optical distribution for brain applications. Based on the results, the best optical source types were determined in each layer. The maximum propagating depth and corresponding source were extracted. The optical source propagating field width was acquired in different depths. The maximum and minimum widths, as well as the corresponding source, were determined. This paper provides a reference for evaluating the optical propagating depth and resolution from an optical simulation aspect, and it has the potential to optimize the performance of optical-based techniques.

## 1. Introduction

Optical-based imaging has developed from early single-location research to further sophisticated imaging in 2D topography and 3D tomography [1]. These techniques have the benefit of high specificity and non-radiative safety for the purposes of diagnosis and therapy, and they have been used in brain imaging applications. Optical imaging, such as functional near-infrared spectroscopy (fNIRS) with lights in and out, has been used for the detection of brain functions [2]. Photoacoustic imaging (PAI), with light in and sound out, has achieved the brain imaging of large and small animals, as well as of human brains [3]. Moreover, optical-based techniques such as optogenetics that are operated with only light into the objects have been used for neural stimulation in animal experiments [4]. However, their performance is limited by complex tissue structures. For instance, the skull and its surrounding layers can lead to signal attenuation and distortion [5]. 

Various optical sources were introduced for different optical simulation needs. Li et al. analyzed the photon penetration depth in a human brain model by applying the Monte Carlo method for light simulation, and this was achieved via treatment with near-infrared light [6]. Two beam types, Gaussian and flat with different beam diameters, were examined in this work. Sharma et al. presented photoacoustic imaging depths under different optical wavelengths using a pencil beam that was based on the Monte Carlo method [7]. Tian et al. studied the spatial resolution and depth sensitivity of optical imaging for absorption or fluorescence-based imaging while using Monte Carlo simulation. A collimated light beam was applied in this research under different numerical apertures and focus [8].

To prompt the performance of optical propagation in brain imaging applications, it is important to determine a suitable optical source. The parameters for defining an optical source include types, size, direction, position, and intensity. Generally, the selected optical source type is determined based on specific applications. There is a lack of systematic studies on the effect of the types of optical sources on brain imaging. When equipment and early experiments were not affordable, modeling and simulation was used instead to explore the optical distribution inside a brain model under different types of optical illumination. Therefore, this paper concerns three geometry categories of optical sources: point source, line source, and surface source [9]. In this work, sixteen optical-source types were used to analyze the optical distribution through brain tissues.

The Monte Carlo simulation method is considered the gold standard of optical simulation in biomedical fields. This method has been widely used in optical coherence tomography (OCT) [10,11], near-infrared spectroscopy (NIRS) [12], fNIRS [13], and PAI [14]. Previously, for optical simulation, we used the diffusion equation [15], which is an approximate method of radiative transfer equation. Based on the hypothesis of the diffusion equation, a scattering coefficient that is much larger than the absorption coefficient is required, which means the optical photons experience enough scattering events before being absorbed [16]. Therefore, the diffusion equation should not be used for optical simulations under matter such as the eyes, cerebrospinal fluid, water, etc. Therefore, we conducted the optical simulation using the Monte Carlo method, which has no limitation with respect to the tissues, and it also delivers high accuracy. 

In this manuscript, we describe the modeling and simulation of different types of optical sources in a simplified brain model based on the Monte Carlo simulation method. We aim to evaluate the propagating depth and resolution based on the optical distribution, as well as seek to determine the optimal optical source for brain imaging applications. Different types of optical source models were built into the proposed simulation model. A 3D human brain simplified model was used in this modeling project to analyze the optical distribution. Also, a quantitative method was utilized to calculate the optical propagating depth and resolution.

## 2. Research Method

### 2.1. Optical Simulation Method

The simulation of the optical fluence (F) and optical absorption (A) was performed in a 3D space with the Monte Carlo method through an open-source MATLAB toolbox named ‘mcxlab’ [17]. The total photon number to be simulated in the study was set at 10^8^. The ending time and time-gate width of the simulation were both set at 5 × 10^−8^ s. The setting of the volume and properties are described in the brain model part. 

In optical-based applications, it is important to characterize the impact of the A of the evaluated medium by considering the photothermal or photoacoustic effect. The A can be calculated based on the absorption coefficient, μ_a_, and the results of the F, as shown in Equation (1) [16],

(1) $$A=\mu_{a}F.$$

### 2.2. Brain Model

**Figure 1:**
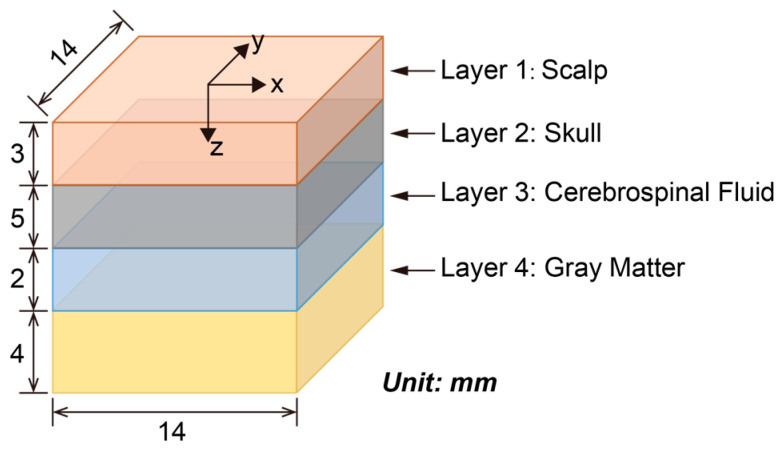
Structure of a simplified brain model with four layers.

The human brain is formed of multi-layer tissues. The brain cortex is protected mainly by the scalp, skull, and cerebrospinal fluid (CSF). In this research project, we defined a four-layer brain model to analyze the optical propagating depth, and distribution with different types of optical sources. The four layers of the brain model include the scalp, skull, CSF, and gray matter, with thicknesses of 3 mm, 5 mm, 2 mm, and 4 mm, respectively [18]. To build a simplified but representative brain model, a 14 × 14 × 14 mm^3^ grid was set as the simulation volume. The thickness of each layer inside this defined volume is shown in Figure 1. The size of each grid was set at 0.1 mm; as such, the volume contained 140 × 140 × 140 grids. We defined the default index of the volume as 0 represent, and index 1 for scalp, index 2 for skull, index 3 for CSF, and index 4 for gray matter. 

The optical properties of the brain tissues under an incident light at 800 nm were used in the optical simulation (listed in Table 1) [19]. By default, there was a set of properties (absorption coefficient = 0.0000 1/mm, scattering coefficient = 0.01/m, anisotropy factor = 1.0000, refractive index = 1) for the background set at the beginning of the optical property array.

### 2.3. Various Types of Optical Sources

The definition of the optical source usually refers to the position, shape, direction, wavelength, intensity, etc. In different simulation models or simulation tools, the optical source’s definition may differ. In our simulation, we used the Monte Carlo method to conduct the optical simulation in MATLAB. The research parameter of the optical source was the shape, so the center position and direction of the optical sources were same in our simulation.

**Figure 2:**
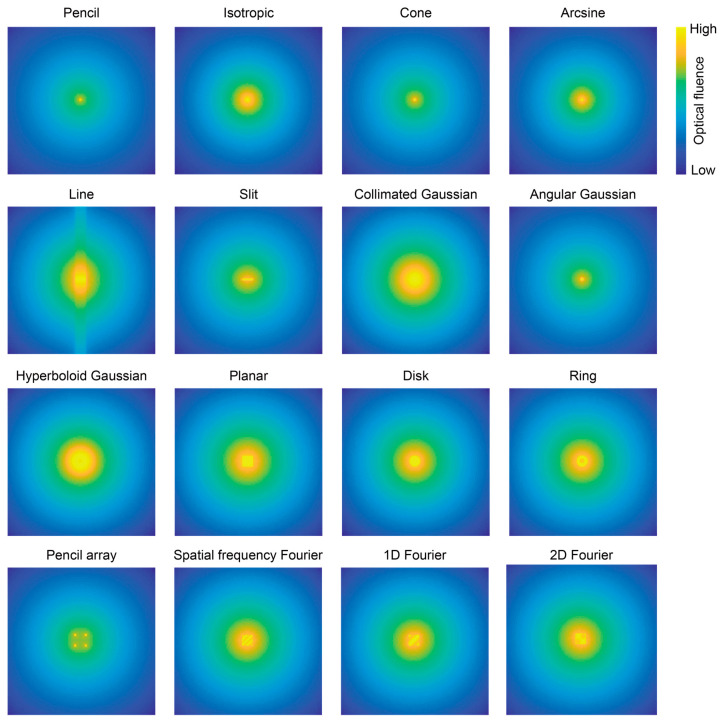
Images of optical fluence distribution of sixteen optical sources at the plane (XY plane at Z = 0.1 mm).

In this research, there were sixteen optical source types adopted for exploration, as shown in Figure 2. Considering the classification of the optical sources with respect to the distribution shape, we divided these optical sources into three geometry categories: point, line, and surface. The point sources launched from an infinitesimal injection point to a propagating direction. From the aspect of the optical energy (E) distribution, the incident positions of the point sources have the maximum energy. Thus, from the energy distribution figure, the point source showed the maximum intensity in the whole area, like a point diffused to surrounding. Four types of point sources were summarized and defined by beams: pencil, isotropic, cone, and arcsine. The line source launched the optical photons from a line region, which included the traditional line source and slit source. The launch region of the surface source was defined in the 2D plane and included collimated Gaussian, angular Gaussian, hyperboloid Gaussian, planar, disk, ring, pencil array, spatial frequency, 1D Fourier, and 2D Fourier types. 

The difference in the optical source types was not only in the distribution shape, but also in the focus and diffusion, as well as uniform and non-uniform distributions. Therefore, the other impact facts, such as the size and diffused angle, were controlled in the same way. The description and definition of the optical sources are summarized in Table 2. All of the light sources were defined at the position (x = 7 mm, y = 7 mm, z = 0 mm), which was considered as the plane of departure. Also, the photon propagated vertically toward the depth-increasing direction. To compare the different optical sources, the number of optical photons used in the simulated models was kept the same.

### 2.4. Propagating Depth and Field Width Quantitation

In this part, we summarize the method, based on the simulation results of F and A, that was used to quantitatively calculate the propagating depth and optical field width.

#### 2.4.1. Data Preprocessing

The E distribution including F and A was acquired from the simulation. Before analyzing the propagating depth and optical field width, the E was normalized from 0 to 100 based on Equation (2),

(2) $$E_{N}=\frac{E_{x,y,z}-E_{\min}}{E_{\max}-E_{\min}}\times 100$$

where E_N_ represents the normalized E (F or A), Ex,y,z named the E at the position (x, y, z), E_max_ donates the maximum value of the E, and E_min_ means the minimum value of the E.

#### 2.4.2. Propagation Evaluation in Each Layer

At the beginning, to understand the number of photons arriving in each layer, optical energy percentage (EP) was used to analyze the optical propagating depth at the layer level. To research what type of optical source makes the maximum value in a specific layer, the EP in each layer was calculated in each model based on the simulation results of F and A, which named as optical fluence percentage (FP) and optical absorption percentage (AP), following Equation (3),

(3) $$P_{i}=\frac{E_{i}}{\sum_{i=1}^{4}E_{i}}$$

where P_i_ represents EP in the i_th_ layer, respectively. E_i_ represents the E like F or A.

In each simulation model, the total EP was 100%. The changes from layer to layer were observed from the EP. The AP was also analyzed as the FP in the same way.

#### 2.4.3. Propagating Depth Evaluation

Firstly, to evaluate the propagating depth, the optical propagating depth was analyzed from the z-axis direction at the center of the XY plane, from where the light source was emitted. The result of the E including F and A was analyzed. 

Secondly, to better understand the change of all source types at Z-axis, the analyzed data in the first step were normalized in this specific range following Equation (4),

(4) $$E_{\mathrm{NZ}}=\frac{E_{\mathrm{xc},\mathrm{yc},z}{-E}_{\min_{\mathrm{xc},\mathrm{yc}}}}{E_{\max_{\mathrm{xc},\mathrm{yc}}}{-E}_{\min_{\mathrm{xc},\mathrm{yc}}}}$$

where E_NZ_ represents the normalized E (F or A), E_xc, yc, z_ named the E at the position (x = 7 mm, y = 7 mm, z), E_max xc, yc_ donates the maximum value of the E at the line of the position (x = 7 mm, y = 7 mm), and E_min xc, yc_ means the minimum value of the E at the line at the position (x = 7 mm, y = 7 mm).

Thirdly, we extracted the maximum depth of each optical source shape under different E levels. Since light E is much lower in deeper regions compared to shallow parts, the best optical source types with maximum depth were determined at three levels: 1%, 0.1%, and 0.01% of the maximum F and A, respectively.

#### 2.4.4. Optical Field Width Evaluation

In this project, we proposed a quantitative method, based on F and A, to evaluate the optical field width from the optical aspect. Optical field width represents the width of the optical illumination field at a certain depth. The optical field width in the X or Y direction was calculated based on the width at half of the maximum height of the E curve, which can also be named as the full width at half maximum (FWHM). 

**Figure 3:**
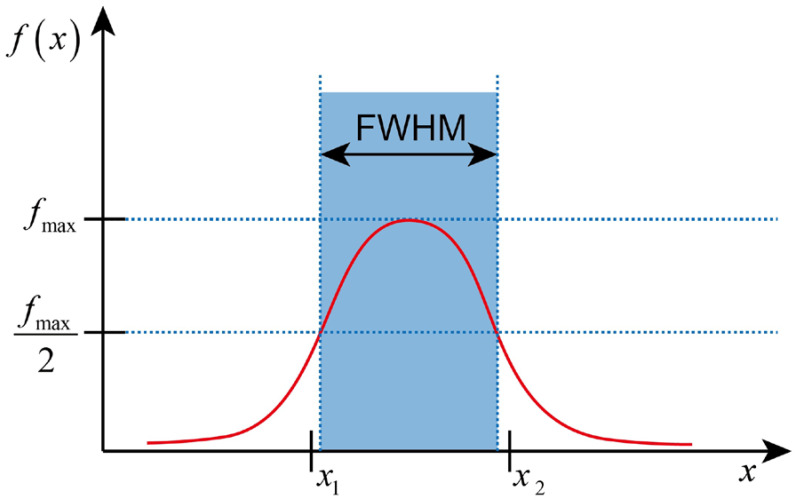
Optical field width evaluation based on the FHWM. The red line represents the intensity distribution function *f*(*x*) of position *x*, while the blue shaded area represents the FWHM region of that distribution.

As shown in Figure 3, it was the extraction of the FWHM. The F or A from the horizontal x-axis and y-axis directions were extracted at a specific depth. The FWHM of the E (F or A) was used to quantitively evaluate the resolution. The peak value of the E in the current direction was determined. Based on the maximum of the E, the full width from the leftmost and rightmost points was computed at the half maximum peak height. Finally, the FWHM was used to represent the optical field width. The FWHM of the E at a depth of 12 mm, was also extracted for comparison to determine the maximum and minimum values, as well as the corresponding source types.

## 3. Results and Discussion

In this section, we analyzed the simulation and quantitative calculated results based on the acquired F and A of the volume. Firstly, we evaluated the FP and AP of each layer. Then, the F and A in the z direction were extracted and discussed. Finally, the F and A in a horizontal direction were researched.

### 3.1. Optical Energy Percentage of Each Layer

**Figure 4:**
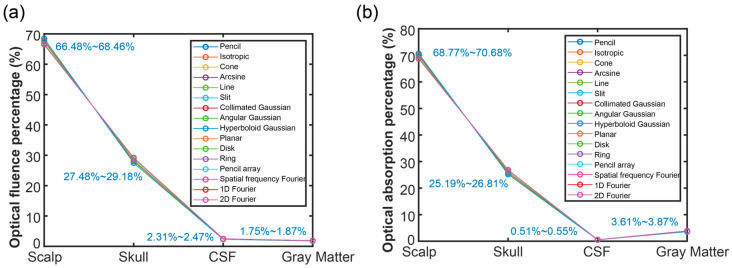
Optical energy percentage in each layer: (**a**) optical fluence percentage in the four layers, and (**b**) optical absorption percentage in four layers when using various optical sources.

Figure 4 shows the FP and AP of each layer when under 16 optical source types. Figure 4a describes the FP of each layer under 16 optical source types. From the figure, the FP gradually decreased following the increase of the tissue depth. The FP was 66.48~68.46% in the scalp layer, 27.48~29.18% in the skull layer, 2.31~2.47% in the CSF layer, and 1.75~1.87% in the fourth layer, the gray matter. The FP was influenced by the distribution of the optical fluence, as well as the tissue thickness. With the known tissue thickness, it can be determined whether the light can arrive at a certain depth for application. According to the results, it can achieve a successful application in the gray matter layer. Still, the performance in the gray matter layer needs to be further analyzed.

The AP of each layer was illustrated in Figure 4b. Following the depth increase, the AP gradually decreased and then increased at the last layer (gray matter). As can be seen in the figure, the AP was 68.77~70.68% in the scalp layer, 25.19~26.81% in the skull layer, 0.51~0.55% in the CSF layer, and 3.61~3.87% in the gray matter layer. Different from FP, the AP in the CSF was smaller than in the gray matter, which is due to the absorption coefficient of the CSF being significantly smaller than that of the gray matter, and the fluence of CSF was not obviously larger than that of the gray matter. Based on the AP, the performance in the related tissues could be predicted better. Due to the surface layer having the most A, the question of deeper optical propagation has become a popular research topic. The key is to see the A intensity in the object so as to determine the application performance.

**Figure 5:**
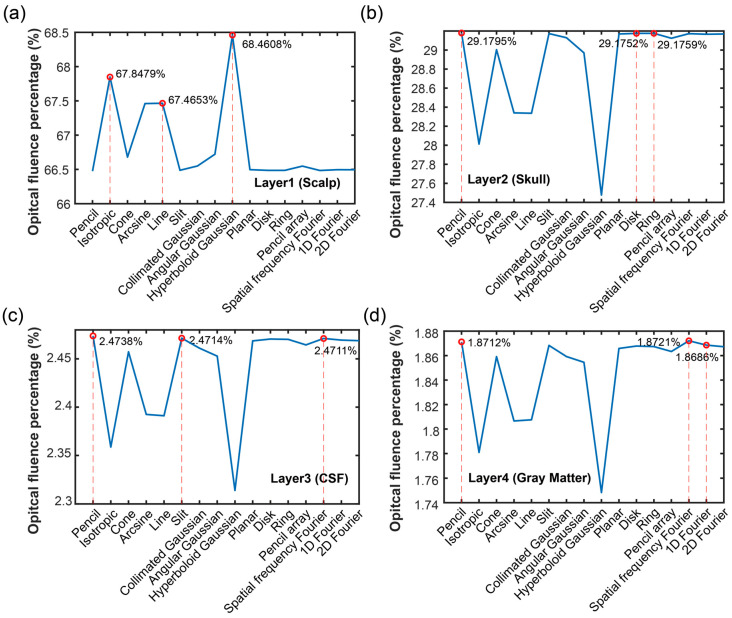
Optical fluence percentages of different optical sources in each layer: (**a**) layer 1 (scalp), (**b**) layer 2 (skull), (**c**) layer 3 (cerebrospinal fluid, CSF), and (**d**) layer 4 (gray matter).

Figure 5 depicts the FP in the four layers using different optical source types, and the top three FPs and their corresponding optical source types were marked. As shown in Figure 5a, the optical source types of the top three FPs in the first layer of the scalp layer were the hyperboloid Gaussian source (68.4608%), isotropic source (67.8479%), and line source (67.4653%). The result was that those optical sources in the surface region had strong diffusion, which made most of the optical photons diffuse and arrive in the first layer. The situation caused fewer optical photons to arrive in the deeper layers; as such, the percentage of the optical fluence of those three optical source types was less than those in the other three layers. In Figure 5b, the optical source types and corresponding value of the top three FPs in the second layer (skull) were pencil source (29.1795%), ring source (29.1759%), and disk source (29.1752%). Based on Figure 5c, the optical source types and their value of the top three FPs in the third layer (CSF) were pencil source (2.4738%), slit source (2.4714%), and disk source (2.4711%). As shown in Figure 5d, in the last layer (gray matter), the optical source types and their value of the top three FPs were spatial frequency Fourier (1.8721%), pencil source (1.8712%), and 1D Fourier (1.8686%). 

Based on these results, it can be deduced that the optical sources had a strong focus and could propagate deeper, which made the FP in the deeper region higher than the other sources. However, the optical source types varied in the different brain layers due to the complex structure and properties of the brain tissues. The optical sources with the top three FPs in the four layers were the isotropic source and pencil beam from the point source category, the line source and slit source of the line source category, and the hyperboloid Gaussian, disk, ring, spatial frequency Fourier and 1D Fourier of the surface source category. Based on the different requirements, the suitable source types could be selected following our results. 

**Figure 6:**
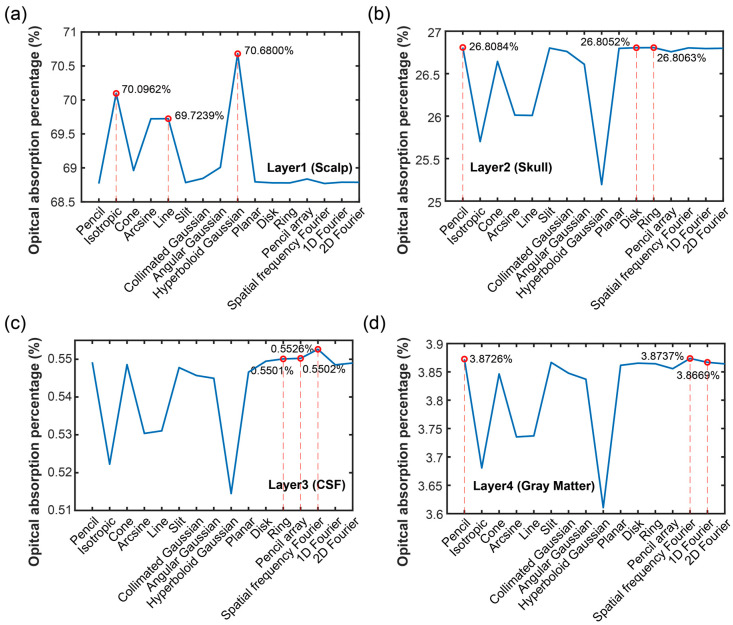
Optical absorption percentages when using different optical sources in each layer: (**a**) layer 1 (scalp), (**b**) layer 2 (skull), (**c**) layer 3 (cerebrospinal fluid, CSF), and (**d**) layer 4 (gray matter).

Considering the optical sorption percentage, the results of the four layers when using different optical source types were shown in Figure 6. As displayed in Figure 6a, the optical source types of the top three APs in the first layer (scalp) were hyperboloid Gaussian source (70.6800%), isotropic source (70.0962%), and line source (69.7239%). In Figure 6b, the optical source type and corresponding value of the top three APs in the second layer (skull) were pencil source (26.8084%), ring source (26.8063%), and disk source (26.8052%). Based on the Figure 6c, the optical source types and their value of the top three FPs in the third layer (CSF) were spatial frequency Fourier source (0.5526%), pencil array source (0.5502%), and ring source (0.5501%). And shown as Figure 6d in the last layer (gray matter), the optical source types and their value of the top three FPs were spatial frequency Fourier source (3.8737%), pencil source (3.8726%), and 1D Fourier source (3.8669%). 

Comparing Figure 5 and Figure 6, it can be seen that the optical source types of the first maximum A in each tissue layer expect for the CSF layer were the same as the first maximum fluence. Based on the previous analysis, we knew that the A in the gray matter immediately increased from the CSF layer due to the different A coefficients in the gray matter and the CSF. The AP decreased in the CSF and then increased in the gray matter following the depth increases. 

### 3.2. Optical Propagation in Vertical Direction

**Figure 7:**
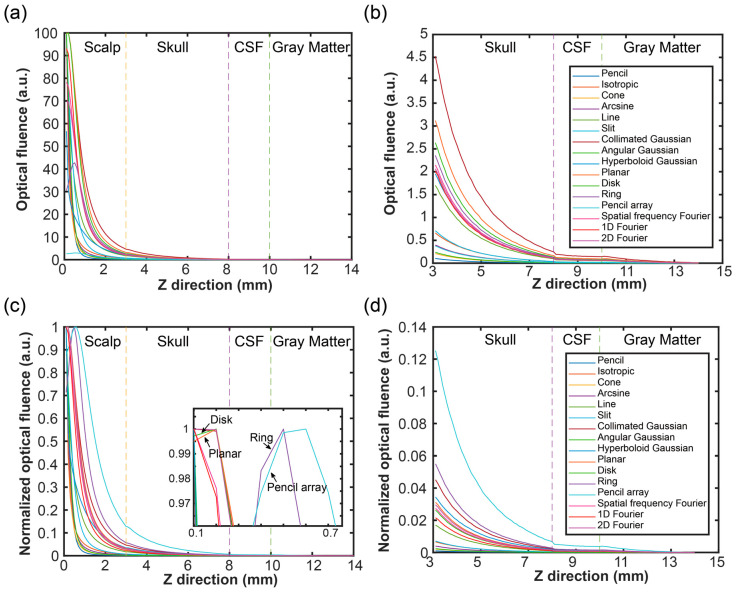
Optical fluence (F) in the Z direction, with an x = 7 mm and y = 7 mm: (**a**) F from the scalp to the gray matter, (**b**) F from the skull to the gray matter, (**c**) normalized F from the scalp to the gray matter, and (**d**) normalized F from the skull to the gray matter.

To evaluate the optical propagating depth, the F and A were extracted from the center position. Figure 7 shows the F of the brain tissues in a depth direction at x = 7 mm and y = 7 mm. As shown in Figure 7a, the change in F in the four tissue layers was determined. The scalp layer had the highest F of all layers. Following a depth increase, the F in the first layer decreased quickly, and then the variations became smooth. The F in the last three layers is shown in Figure 7b. The results indicated that the F still decreased following the depth increase. In addition, at the boundary of each layer, the sudden change was obvious. From the skull layer to the CSF layer, the F suddenly decreased. Meanwhile, the F increased a bit from the CSF layer to the gray matter layer.

To distinguish the difference of each source, the normalized F in the z direction was applied in Figure 7c,d. As shown in Figure 7c, the change in F in the four tissue layers was determined. In the surface region (the first layer), there were evident differences compared to the results shown in Figure 7a. Through optical scattering and absorption, the F in the first layer became consistent and decreased following depth increase. The F with different initial values in the last three layers, decreased following the depth increase while compared to those in Figure 7d.

Meanwhile, after zooming Figure 7c, the disk source, planar source, ring source, and pencil array beam all increased first and then decreased in the surface region. The disk source and planar source were like each other, while the ring source and pencil array source were similar to each other. The reason for this was that the disk source and planar source were uniform sources in the initial position, which made their F similar to each other. The ring source and pencil array source had no optical photons emitted from the center of the initial position, which resulted in the lowest degree of F in the center of the initial plane. Thus, when optical photons completely diffused in the surface region and overlay, the F first increased and then decreased.

**Figure 8:**
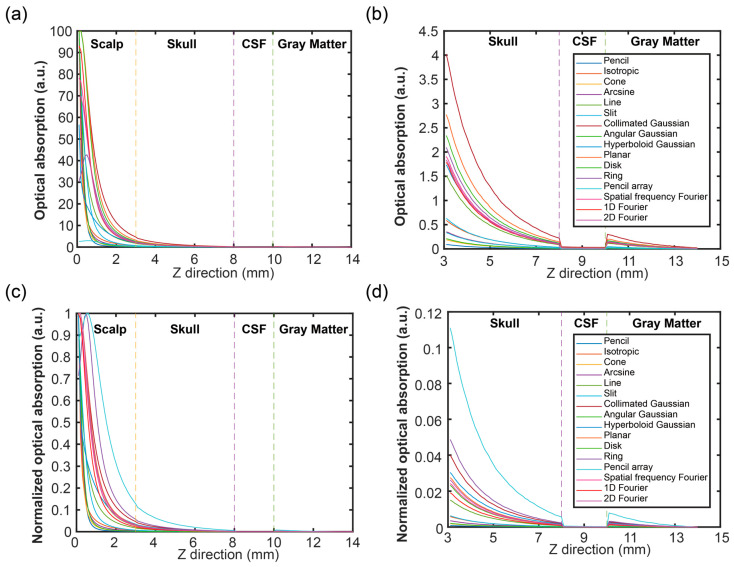
Optical absorption (A) in the Z direction, with an x = 7 mm and a y = 7 mm: (**a**) A from the scalp to the gray matter, (**b**) A from the skull to the gray matter, (**c**) normalized A from the scalp to the gray matter, and (**d**) normalized A from the skull to the gray matter.

The A of the brain tissues among a depth direction of x = 7 mm and y = 7 mm was depicted in Figure 8. Figure 8a,b show the results of the A without normalization in an in-depth direction. As displayed in Figure 8a, the A in the scalp layer, which was same as the F, had the highest value. As shown in Figure 8b, the A with different levels in each layer of the skull, CSF, and gray matter still decreased following the depth becoming deeper. In addition, the A nearly changed to 0 in the CSF layer. Then, at the beginning of the gray matter layer, the A had a gradient increase from the CSF layer, then decreased following the depth increases. The reason for this was that the A coefficient of the CSF was much smaller than the gray matter; so, the gradient increase was evident.

Figure 8c,d depict the results of the normalized A in an in-depth direction. According to Figure 8c, the characteristics of the normalized A were magnified in the same manner as the F. In addition, based on Figure 8d, the A was significantly lower in the CSF layer than other tissues. Due to the lower A of the CSF and the higher absorption coefficient of the gray matter than the other matters, this phenomenon in the last three layers with normalized A became more obvious than those without normalized A.

**Figure 9:**
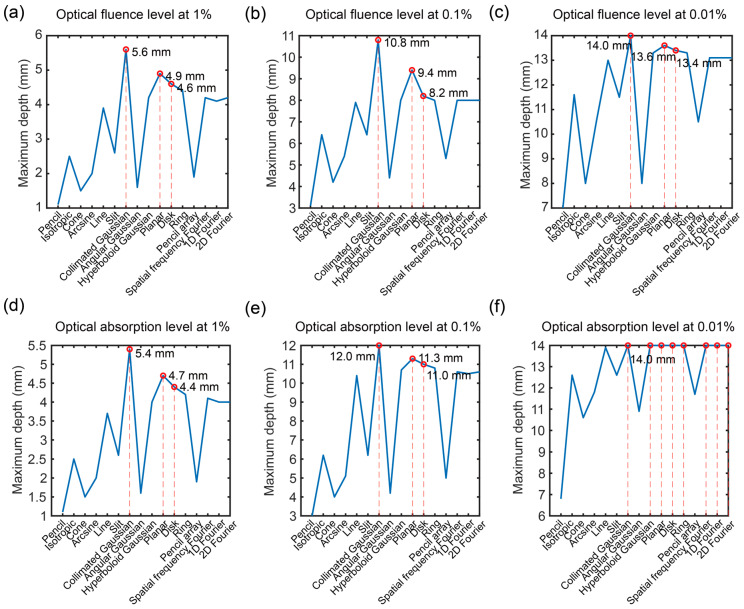
Maximum depth at specific optical energy levels: optical fluence (F) level of (**a**) 1%, (**b**) 0.1%, and (**c**) 0.01%; and optical absorption (A) level of (**d**) 1%, (**e**) 0.1%, and (**f**) 0.01%.

To compare different optical source types, the results of the maximum depth at specific E levels (1%, 0.1%, and 0.01%) were shown in Figure 9. Based on Figure 9a, when the F level was at 1%, the first three maximum depths were 5.6 mm via collimated Gaussian source, 4.9 mm via planar source, and 4.6 mm via disk source. As shown in Figure 9b, the first three maximum depths were 10.8 mm via collimated Gaussian source, 9.4 mm via planar source, and 8.2 mm via disk source when the F level was at 0.1%. In Figure 9c, when the F level was at 0.01%, the first three maximum depths were 14.0 mm via collimated Gaussian source, 13.6 mm via planar source, and 13.4 mm via disk source. From these results, the collimated Gaussian source demonstrated the maximum depth in three of the different F levels for deep propagation.

Figure 9d–f show the results of the maximum depth at specific A levels (1%, 0.1%, and 0.01%). According to Figure 9d, when the A level was at 1%, the first three maximum depths were 5.4 mm via collimated Gaussian source, 4.7 mm via planar source, and 4.4 mm via disk source. As shown in Figure 9e, the first three maximum depths were 12.0 mm via collimated Gaussian source, 11.3 mm via planar source, and 11.0 mm via disk source, while the F level was at 0.1%. Based on Figure 9f, when the F level was at 0.01%, the first three maximum depths were 14.0 mm via collimated Gaussian, hyperboloid Gaussian, planar, disk, ring, spatial frequency Fourier, 1D Fourier and 2D Fourier sources. Here, the A’s results were different from the F’s results at the level of 0.01%. This is because the A of the gray matter layer was higher than the layer in the CSF. From these results, the collimated Gaussian source method demonstrated the maximum depth in those three A levels for deep propagation.

### 3.3. Optical Distribution in a Horizontal Direction

**Figure 10:**
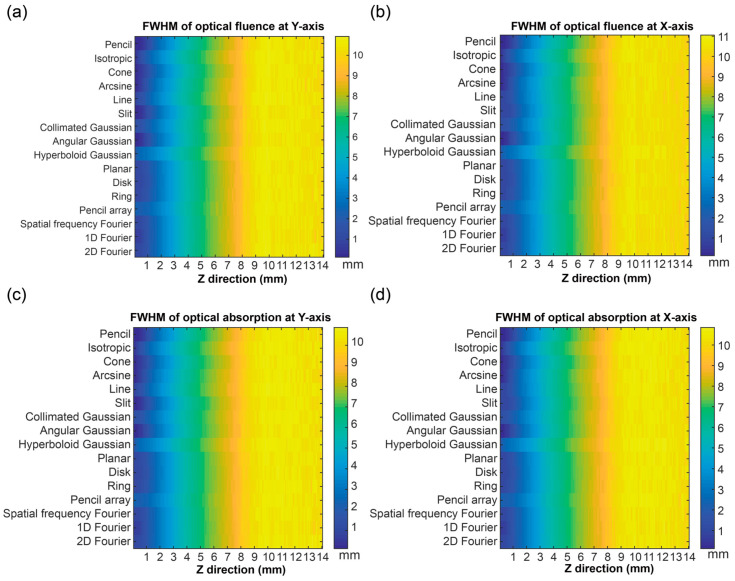
The full width at half maximum (FWHM) of the optical fluence (F) and optical absorption (A) in an X-axis direction and Y-axis direction: the FWHM of the F in (**a**) a Y-axis direction and (**b**) an X-axis direction; and the FWHM of the A in (**c**) a Y-axis direction and (**d**) an X-axis direction.

To analyze the optical distribution in the horizontal direction, FWHM was used to represent the optical field width of the results. Figure 10 shows the FWHM of the F and A in the x direction and y direction in all depth grids. The results showed that the FWHM became larger in value with the depth increases. The FWHM of different optical sources were different in the surface region. The FWHM of those sources gradually showed less evident difference in the deeper region. Based on these results, the pencil beam, cone beam, angular Gaussian beam, and spatial frequency Fourier source had the smaller FWHMs, while the others were relatively larger.

**Figure 11:**
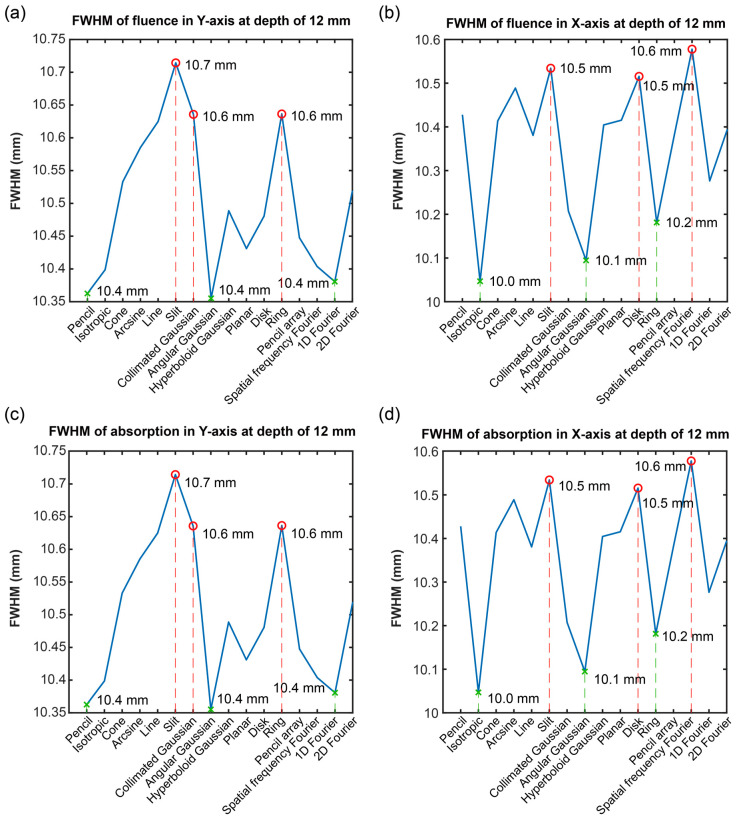
The full width at half maximum (FWHM) of the normalized optical fluence (F) and optical absorption (A) in an X-axis direction and Y-axis direction at a depth of 12 mm: the FWHM of the F in (**a**) a Y-axis direction and (**b**) an X-axis direction; and the FWHM of the A in (**c**) a Y-axis direction and (**d**) an X-axis direction.

Due to the difficulty distinguishing between the data information under specific depths in Figure 10, Figure 11 exhibits the results of the FWHM in the Y and X axes at the depth of 12 mm. After comparison, it was found that the F and A had similar FWHM data. The reason for this was that the FWHM was the parameter in the E distribution but not in the E intensity level; thus demonstrating that the diffusion of the F and A was the same in the horizontal direction. The difference in the FWHM was from the direction of the Y-axis and X-axis, which can be found through the difference of the FWHM in the Y-axis and X-axis.

From the results of Figure 11a,c, the first three maximums of the FWHM in the Y-axis were shown in the slit, collimated Gaussian, and ring sources, which thus illustrated that those types of optical sources had larger diffusions. In addition, the first three minimums of the FWHM in the Y-axis were shown in the pencil array, angular Gaussian and 1D Fourier sources, which thus illustrated that those types of optical sources had less diffusions. 

According to Figure 11b,d, the first three maximum values of the FWHM in the X-axis were shown in the spatial frequency Fourier, slit, and disk sources, which thus illustrates that those types of optical sources had larger diffusions. Meanwhile, the first three minimums of the FWHM in the X-axis were shown in the isotropic, angular Gaussian, and ring sources, which thus illustrates that those types of optical sources had less diffusions.

### 3.4. Discussion

Based on the research, it was found that most of the research was regarding a few specific optical source types. The literature lacks systematic research on the influence of different optical source types on optical distribution in brain tissues. Therefore, sixteen optical sources according to distribution shape were classified into three categories, point, line, and surface, for the purpose of this research. Meanwhile, the optical source types were defined based on the distribution shape, focus and diffusion, as well as uniform and non-uniform distributions.

The point sources included the pencil beam, isotropic source, cone beam, and arcsine source methods. The pencil beam, cone beam, and arcsine source methods had good focus inside the point sources; the focus of the pencil beam was the best. The other point source—the isotropic source—had the largest diffusion out of all the point source methods. 

The line sources included a diffuse line source and slit source. The diffuse line source had a good focus in one horizontal direction, as well as good diffusion in another horizontal direction. And the slit source had a good focus in all horizontal directions. The line source was not often used except in special conditions.

The surface sources included the planar, disk, ring, angular Gaussian, collimated Gaussian, hyperboloid Gaussian, pencil array, spatial frequency, 1D Fourier, and 2D Fourier source methods. The planar, disk, and ring sources belonged to the uniform surface source, and they had the same focus and diffusion but with different types. The rest of the types belonged to the nonuniform surface source approaches. 

The angular Gaussian and the collimated Gaussian sources were the most like the point source methods, but they were distributed in the surface plane instead. Compared with the point source approaches, the collimated Gaussian method had a better penetration depth under the same scale as the normalized F rate in terms of volume. 

Based on the references, pencil beam [7], collimated Gaussian [6], and planar source [6] methods are the approaches that are often used in simulation work, which is consistent with our research results. In addition, based on the different requirements, other optical source types can be used for specific research. For example, the isotropic source is representative of self-luminous or radiating objects. A cone source can also be added for the diffusion source with a certain angle.

### 3.5. Limitations and Prospects

In this manuscript, we focused on the research of different types of optical sources in a simplified brain model based on the Monte Carlo simulation method. There are still some limitations to the research. The topics that need further exploration are presented.

(1) Distribution size of the sources. Although the parameters of the optical sources are set as consistently as possible in this research, there is an obvious difference in the distribution size of the point, line, and surface sources. This phenomenon is due to the definition of the initial launch distribution and other settings of those source types. Therefore, the distribution size of line and surface sources can be researched and compared with point sources.
(2) Increasing source number and intensity. In this manuscript, we found that the difference in propagating depth and FWHM of the different optical source types at the deep region is not as strong as in the surface region. Further, increasing source number and intensity can be researched for deep imaging under optical safety standards.
(3) Experiment validation. The experimental validation allows us to validate the simulation model in real scenarios. However, due to the complex human brain tissues, in this paper we focused on the simulation exploration of the optical source types. The experiment validation can be conducted following the systematical analysis of the optical source.
(4) Tissue uncertainty. Not only the properties of the tissues can impact the simulation research, but also the thickness and the distribution of each layer. The uncertainty of tissue is a very interesting topic to explore. This work only focused on one brain model with a referred property set. It is expected to research on the more complex brain model with variability.

## 4. Conclusions

In this research, the optical simulation—based on the Monte Carlo method—of different optical source types in the brain was conducted to evaluate the propagating depth and resolution, which were based on optical distributions, so as to determine the optimal type of optical source for brain imaging applications. There were four main kinds of optical sources, including sixteen types of optical sources, studied. 

Firstly, to understand the number of photons arriving in layers, the FP of each layer was analyzed based on the simulation with different optical sources; the analysis showed that more than 1% of FP arrived in the gray matter layer, which means that the optical photons can interact with gray matter for applications. The optical source types that delivered maximum FP were determined. The optical source types of the top three FPs in the first layer (scalp layer) were hyperboloid Gaussian source, isotropic source, and line source. The optical source types and corresponding value of the top three APs in the second layer (skull) were pencil source, disk source, and ring source. In the third layer (CSF), the optical source types and their value of the top three FPs were spatial frequency Fourier source, pencil array source, and ring source. In the last layer (gray matter), the optical source types and their value of the top three FPs were spatial frequency Fourier source, pencil source, and 1D Fourier source.

To evaluate the optical propagating depth, the change in the F and A in the z direction were discussed. The first three maximum depth and corresponding optical source types were determined when under three levels of the maximum F (1%, 0.1%, and 0.01%). The collimated Gaussian source demonstrated the maximum depth in three different A levels with respect to deep propagation, and the other two tops were planar and disk sources.

For the optical propagating resolution analysis, the FWHM of the F and A were computed. The F and A had similar FWHMs in all the data. However, the FWHM in the Y direction and X direction was different. In summary, at the depth of 12 mm, the optical sources with the largest diffusion were the slit, collimated Gaussian, and ring sources on the Y-axis; likewise, spatial frequency Fourier, slit, and disk sources had the largest diffusion on the X-axis. The optical sources with the least diffusions were pencil, angular Gaussian, and 1D Fourier sources on the Y-axis, and isotropic, angular Gaussian and ring sources were the ones with the least diffusions on the X-axis.

The present work gives an overview of the different optical source types, as well as providing a reference for evaluating the optical propagating depth and field width from the optical simulation to determine the performance of optical source selection for the purpose of system design. Although the difference of different optical source types looks to not be strong enough, the difference will accumulate with the increase of parameters such as size, number, and intensity of the optical sources. After determining the optical source types, more exploration of the optical sources for optical-based techniques in the human brain is expected, such as studying the incident energy, number, and distribution of the optical sources. The incident energy would be the next research topic to determine the input energy and optical photons for simulation as they can also influence the number and distribution of the source settings.

## Figures and Tables

**Table 1:** Optical properties of the brain tissues at the optical wavelength of 800 nm.

Tissue Layers	Absorption Coefficient, μ_a_ (1/mm)	Scattering Coefficient, μ_s_ (1/mm)	Anisotropy Factor, g	Refractive Index, n
Scalp	0.018	19.0	0.9	1.37
Skull	0.016	16.0	0.9	1.43
Cerebrospinal fluid	0.004	2.4	0.9	1.33
Gray matter	0.036	22.0	0.9	1.37

**Table 2:** Description of the different categories of the optical source types.

No.	Source Type	Category	Characteristics	Parameter ^a^
1	Pencil beam [20]	Point	Highly directional and focused with a narrow beam profile.	NA
2	Isotropic beam [9]	Point	Radiates equally in all directions with a spherical distribution.	NA
3	Cone beam [21]	Point	Uniformly expands in a conical shape.	Half-angle in radian: π/6
4	Arcsine [22]	Point	Illuminates uniformly over a wide viewing angle with a cosine-squared distribution.	NA
5	Collimated Gaussian [23]	Surface	Bell-shaped intensity profile and uniform over a long distance; it is a parallel, collimated beam that is created by a collimating lens from a Gaussian beam.	Waist radius: 10
6	Angular Gaussian [24]	Surface	Emits in a Gaussian distribution over a specific zenith angle.	Zenith angle: π/6
7	Hyperboloid Gaussian [25]	Surface	Illuminates in a hyperboloid shape with Gaussian distributions, and it has differences in horizontal and vertical distributions.	Waist radius: 10 Distance between launch plane and focus: 1 Rayleigh range: π/6
8	Line [26]	Line	Emits uniformly from the line region into the perpendicular direction.	Length: 10
9	Slit [27]	Line	Emits with a collimated beam from the line region.	Length in X direction: 10
10	Planar [9]	Surface	Illuminates uniformly from a 3D quadrilateral planar distribution.	Length in X and Y directions: 10
11	Disk [28]	Surface	Illuminates uniformly from a 3D disk distribution pointing along the source direction.	Radius: 5
12	Ring [29]	Surface	Illuminates uniformly from a 3D ring distribution, and it points along the source direction.	Outer radius: 5 Inner radius: 1
13	Pencil array [30]	Surface	Distributes as a rectangular array of pencil beams.	Length in X and Y directions: 10 2 × 2 pencil beams
14	Spatial Frequency Fourier [31]	Surface	Illuminates in a spatial frequency domain distribution in a planar shape.	Length in X and Y directions: 10 X/Y frequencies: 2/2
15	1D Fourier [32]	Surface	Emits light in a general Fourier distribution in the x direction.	[v1x, v1y, v1z, |v2|] = [10, 0, 0, 10] ^b^ [kx, ky, phi0, M] = [1, 1, 0, 0] ^b^
16	2D Fourier [32]	Surface	Emits light in a general 2D Fourier distribution.	[v1x, v1y, v1z, |v2|] = [10, 0, 0, 10] ^b^ [kx, ky, phi0, M] = [1, 1, 0, 0] ^b^

^a^ The unit of the length in this table was shown in the grid size in 3D voxels. It can be converted into a millimeter with a ratio of 0.1 mm. ^b^ More details about the source setting can be found in the main function of “mcxlab” from the MATLAB toolbox.

## Data Availability

The datasets generated for this study are available on request to the corresponding authors.

## List of Abbreviations

1D	One dimensional
2D	Two dimensional
3D	Three dimensional
A	Optical absorption
AP	Optical absorption percentage
CSF	Cerebrospinal fluid
E	Optical energy
E_i_	Optical energy like optical fluence or absorption
E_max_	Maximum value of the energy
E_max xc, yc_	Maximum value of the energy at the line of the position (x = 7 mm, y = 7 mm)
E_min_	Minimum value of the energy
E_min xc, yc_	Minimum value of the energy at the line at the position (x = 7 mm, y = 7 mm)
E_N_	Normalized energy (optical fluence or absorption)
E_NZ_	Normalized energy (optical fluence or absorption)
E_x,y,z_	Energy at the position (x, y, z)
E_xc, yc, z_	Energy at the position (x = 7 mm, y = 7 mm, z)
EP	Optical energy percentage
F	Optical fluence
fNIRS	Functional near-infrared spectroscopy
FP	Optical fluence percentage
FWHM	Full width at half maximum
g	Anisotropy factor
n	Refractive index
NA	Not applicable
NIRS	Near-infrared spectroscopy
OCT	Optical coherence tomography
PAI	Photoacoustic imaging
P_i_	Optical energy percentage in the i_th_ layer
μ_a_	Absorption coefficient
μ_s_	Scattering coefficient
